# Mediating and moderating effects of plasma proteomic biomarkers on the association between poor oral health problems and brain white matter microstructural integrity: the UK Biobank study

**DOI:** 10.1038/s41380-024-02678-3

**Published:** 2024-07-30

**Authors:** May A. Beydoun, Hind A. Beydoun, Yi-Han Hu, Zhiguang Li, Michael F. Georgescu, Nicole Noren Hooten, Mustapha Bouhrara, Jordan Weiss, Lenore J. Launer, Michele K. Evans, Alan B. Zonderman

**Affiliations:** 1 Laboratory of Epidemiology and Population Sciences, National Institute on Aging, NIA/NIH/IRP, Baltimore, MD 21224 USA; 2 VA National Center on Homelessness Among Veterans, U.S. Department of Veterans Affairs, Washington, DC 20420 USA; 3 Department of Management, Policy, and Community Health, School of Public Health, University of Texas Health Science Center at Houston, Houston, TX 77030 USA; 4 Laboratory of Clinical Investigation, National Institute on Aging, NIA/NIH/IRP, Baltimore, MD 21224 USA; 5 Stanford Center on Longevity, Stanford University, Stanford, CA 94301 USA

**Keywords:** Neuroscience, Biological techniques, Molecular biology, Predictive markers

## Abstract

The plasma proteome can mediate associations between periodontal disease (Pd) and brain white matter integrity (WMI). We screened 5089 UK Biobank participants aged 40–70 years for poor oral health problems (POHP). We examined the association between POHP and WMI (fractional anisotropy (FA), mean diffusivity (MD), Intracellular Volume Fraction (ICVF), Isotropic Volume Fraction (ISOVF) and Orientation Diffusion (OD)), decomposing the total effect through the plasma proteome of 1463 proteins into pure mediation, pure interaction, neither, while adjusting for socio-demographic and cardiovascular health factors. Similarly, structural equations modeling (SEM) was conducted. POHP was more prevalent among men (12.3% vs. 9.6%), and was associated with lower WMI on most metrics, in a sex-specific manner. Of 15 proteins strongly associated with POHP, growth differentiation factor 15 (GDF15) and WAP four-disulfide core domain 2 (WFDC2; also known as human epididymis protein 4; HE4) were consistent mediators. Both proteins mediated 7–8% of total POHP effect on FA_mean_. SEM yielded significant total effects for FA_mean_, MD_mean_ and ISOVF_mean_ in full models, with %mediated by common latent factor (GDF15 and WFDC2) ranging between 13% (FA_mean_) and 19% (ISOVF_mean_). For FA, mediation by this common factor was found for 16 of 49 tract-specific and global mean metrics. Protein metabolism, immune system, and signal transduction were the most common pathways for mediational effects. POHP was associated with poorer WMI, which was partially mediated by GDF15 and WFDC2.

## Introduction

Dementia is a leading cause of death and disability and its impact on population health is expected to rise in response to population aging [1, 2]. Risk factors for Alzheimer’s disease (AD)—the leading cause of dementia [2, 3]—include older age, family history, and genetics, with the APOE4 allele being the main genetic risk factor [2]. The 2020 Lancet commission identified 40% of dementia cases as attributed to 12 modifiable risk factors that at midlife may have an impact on the emergence of neuropathology and have been associated with early brain markers of neurodegeneration [4].

Recent studies identified systemic and CNS-based infectious pathogens as potential risk factors for AD and other forms of neurodegenerative diseases. Infectious agents are hypothesized to cause AD through CNS infiltration, severe systemic inflammation, or by inducing autoimmunity in response to certain pathogens. The polymicrobial hypothesis has been proposed as a more logical process for AD pathogenesis [5]. Periodontal bacteria (*Porphyromonas gingivalis* (*P. gingivalis*), *Tannerella forsythia* (*T. forsythia*), *Aggregatibacter actinomycetemcomitans* (*A. actinomycetemcomitans*), Treponema denticola (*T. denticola*)) are responsible for periodontal disease (Pd) [6], which can result in gingival inflammation, connective tissue deterioration, periodontal pocket development, alveolar bone loss, and tooth loss. A recent investigation found that *P. gingivalis* and resulting gingipains in the brain play a critical role in AD etiology and that Aβ1-42 is produced in the brain partly as a reaction to this infection. Epidemiological data connecting Pd-related illnesses, including *P. gingivalis*, to AD or early markers of neurodegeneration associated with AD, however, is scarce.

Magnetic resonance imaging (MRI) is a useful tool for identifying and quantifying vascular brain injuries and structural abnormalities. Diffusion-Weighted Imaging (DWI) measures tissue-level water diffusion rate, detecting water movement within tissue architecture [7]. DTI indices provide quantitative probes of the magnitude and directionality of water molecules. Fractional anisotropy (FA) reflects the directionality of molecular displacement by diffusion, with values between 0 and 1; higher values are an indicator of anisotropic diffusion that confers a preferred direction, whereas mean diffusivity (MD) reflects the average magnitude of molecular displacement by diffusion, with higher values indicating more freely diffusing water [7, 8]. FA and MD are proxies of neuronal status, including axonal, myelin, dendritic, and synaptic integrity.

Although DTI-indices are sensitive to capture early changes in tissue microstructure, they are lacking specificity to different diffusion tissue compartments. To overcome this difficulty, the neurite orientation dispersion and density imaging (NODDI) MRI technique has been introduced [9, 10]. Indeed, studies have shown that NODDI derived outcomes are more sensitive and specific to clinical outcomes and target pathologies as compared to DTI-indices [9, 10]. Indeed, NODDI provides measures of the orientation dispersion index (OD or ODI), intracellular volume fraction (ICVF), generally defined as the neurite density index (NDI), and volume fraction of the isotropically diffusing water (ISOVF) [11]. NDI/ICVF reflects the intrinsic water molecules transport within the axons or neurites, ISOVF provides a measure of the freely-diffusing water, while OD indicates the dispersion extent of the neurites or axons [11]. Both ISOVF and OD are expected to be negatively correlated with brain health, whereas ICVF integrity, is expected to be positively correlated with brain health with higher values indicating greater neurite density or integrity [11]. Recent studies have shown that POHP or specifically Pd, were associated with poorer WMI and other brain MRI markers of the dementia phenome [12, 13]. Studies suggest that the plasma proteome may be connected to all-cause dementia [14–19], though little is known about whether the proteome may be influenced by Pd in relation to early neuroimaging markers of dementia [9, 20].

The associations of Pd and poor oral health problems (POHP) with MRI-based markers of dementia remain underexplored. We examined associations of POHP with several dementia phenome-related brain MRI DTI and NODDI metrics of WMI among dementia-free older adults, focusing on mediation through the plasma proteome, and using data from the UK Biobank.

## Methods

### Database

The UK Biobank surveyed more than 500,000 UK residents between the ages of 37 and 73 over the period of 2006 through 2010 [21]. Study details are discussed elsewhere [21]. Participants were evaluated by qualified staff who collected biological samples and phenotypic measurements [22]. The Northwest Multi-Centre Research Ethics Committee approved the UK Biobank. The Institutional Review Board of the National Institutes of Health and the UK Biobank access management team both gave their consent to this project as part of application #77963.

### Study sample

**Fig. 1:**
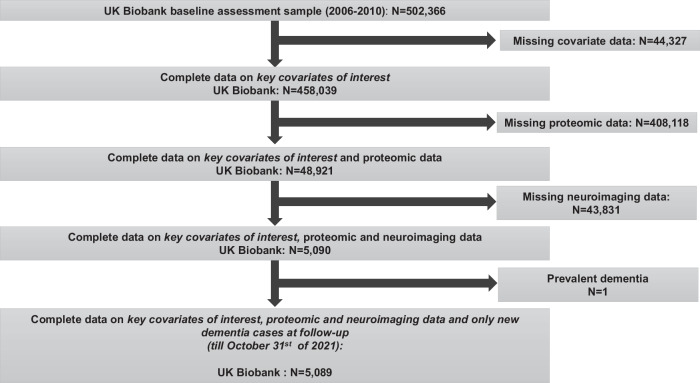
Participant flowchart. UK United Kingdom.

Figure 1 depicts the participant flowchart starting from the initial sample of 502,366 UK Biobank participants, of whom only 5089 had neuroimaging data, other key covariates of interest and proteomic data measured at baseline assessment (2006–2010), and who were also dementia-free at baseline.

### Brain MRI acquisition and processing

Brain MRIs were performed on a subset of UK Biobank participants in an ongoing ancillary study (45k participants up to October of 2022; initiated in 2014) at three MRI locations spread across the study area [23, 24]. All brain MRI data were acquired using similar 3T Siemens Skyra scanners, according to a freely available protocol (http://www.fmrib.ox.ac.uk/ukbiobank/protocol/V4_23092014.pdf), with documentation and published methods, including the image processing and QC pipeline, available at: http://biobank.ctsu.ox.ac.uk/crystal/docs/brain_mri.pdf.

Briefly, the UK biobank team analyzed white matter tract-averaged diffusion MRI (dMRI) indices and made them available to approved researchers as imaging-derived phenotypes (IDPs), which included the DTI and NODDI metrics (see Online Supplementary Methods (OSM) 1 for more details). Here, we limited the IDPs to the available DTI and NODDI measures, including FA, MD, ISOVF, ICVF, and ODI across white matter tracts [25]. The key outcomes of interest were tract-averaged global means of FA, MD, ISOVF, ICVF and OD, with tract-specific values deemed secondary outcomes of relevance, and details provided in Online Supplementary Methods (OSM) 1.

### Poor oral health problems, POHP

POHP (yes = 1 vs. no = 0) were derived from self-report indicating “loose teeth” or “dentures” or diagnosed Pd included UKB field code 131562 (“Date K05 first reported (gingivitis and periodontal diseases)), including all sources of data (e.g. outpatient and hospitalization) as long as those occurred prior to baseline assessment. Report of both loose teeth and denture was excluded as unexposed, given that partial loose teeth with denture may be considered as “treated” severe oral health problem, while “denture only” is considered a severe advanced stage with potential for complete edentulation. The two self-report questions were chosen due to their strong genetic correlation with periodontitis and tooth loss, respectively [26]. This main exposure and its rationale is comparable to the one used by another study with similar objectives, excluding proteomic analyses [27]. A sensitivity analysis for association with the key outcome FA_mean_ was carried out on three alternative exposure definitions: (i) “severe reported health problems including both dentures and loose teeth: 0 = none, 1 = one and 2 = both”, irrespective of diagnosis; (ii) “ diagnosed prevalent periodontal disease, yes vs. no”; and (iii) all self-reported oral problems (yes vs. no), including loose teeth, dentures, mouth ulcers, toothache, painful gums and bleeding gums, irrespective of diagnosis; (iv) proxy measure for self-reported Pd, as described by previous studies, which included self-reported painful gums, bleeding gums and/or having loose teeth [28].

### Olink proteomics

Olink® Explore 1536 Proteomics platform was used for proteome analysis of 54,306 plasma samples from distinct UK Biobank participant-visits as part of the UK Biobank Pharma Proteomics Project (UKB-PPP), 1472 protein analytes—corresponding to 1463 distinct proteins—from panels for inflammation, cancer, cardiometabolic, and neurological conditions—were measured using Proximity Extension Assay (PEA) technology. Sun et al. provides information on the PEA tests, normalization, and pre-processing of the data [29]. Normalized protein expression (NPX) units on a log2 scale are used, to represent protein data [29]. Since the proteins are already Log2 transformed and normally distributed, we have only modified them to standardized z-scores within the final largest selected sample.

#### Covariates

We included age, sex, race/ethnicity (White, Black, South Asian, and Others), and household size as well as the Townsend deprivation index (TDI), pre-tax household income (1 = “£18,000,” 2 = “£18,000–£29,999,” 3 = “£30,000–£51,999,” 4 = “£52,000–£100,000,” and 5 = “ > £100,000), and educational accomplishment (0 = Low, combining “None,” “CSEs/Equivalent,” “NVQ/HND/HNC/Equivalent,” and “Other professional qual”; 1 = Intermediate, combining “O Levels/GCSEs/Equivalent,” and “A/AS Levels Equivalent”; and 2 = Higher level) [30]. TDI ratings were computed using national census data, accounting for residential postcode-level car ownership, crowded housing, owner occupation, and unemployment. A higher TDI score denotes socioeconomic deprivation [31]. To reflect higher socioeconomic status (SES), TDI was multiplied by −1 and mixed with z-scores for household income and educational attainment to create a single SES summary score. Potential confounding variables included a composite measure of cardiovascular health, known as the American Heart Association’s “Life’s Essential 8” (LE8). Supplementary Method 2 and Supplementary Tables 1–3 include specifics about this measure.

### Statistical analysis

Stata 18.0 (StataCorp, College Station, Texas) was used for all analyses, while data visualization was partly accomplished using R version 4.3.1. The overall and sex stratified sample’s means and proportions of important study characteristics were determined. Additionally, the same estimates were contrasted by sex using multinomial, logistic, and linear logit models. To investigate the adjusted relationships of POHP exposure on the various WMI parameters, multiple linear regression models were used, adjusting for baseline age, sex, race, SES, household size and LE8 total score. In most analyses where sex was the main stratifying factor, regression models were conducted on the total sample as well as separately on men and women. With simultaneous correction for the same covariates (Stata *parmby*, *qqval*, and *multproc* commands), POHP was included to a series of distinct multiple linear regression models, with outcomes alternating among 1463 plasma proteomic biomarkers. To display the p-values and effect sizes of each of the 1463 equations and to highlight those that passed multiple testing with the Bonferroni adjustment, a volcano plot was made using the R *ggplot* package. The top hits with >0.25 effect size in absolute value (corresponding to ¼ SD higher plasma protein value among the POPH^+^ group vs. POHP^−^) were selected among those who passed Bonferroni correction. This effect size is considered to be weak to moderate based on several guidelines (https://cran.r-project.org/web/packages/effectsize/vignettes/interpret.html). A weaker effect size threshold was chosen if needed, corresponding the top 99th percentile (or 1st and 99th percentile on either side), with the goal of reducing the number of selected proteins to <30 for this part of the analysis. A similar approach was applied previously in comparable studies testing plasma proteomics from the UK Biobank against other exposures [32–34].

These selected biomarkers were then included in a four-way decomposition model with one of each selected proteomic marker as the mediator, POHP as the primary exposure, FA_mean_ as the primary outcome (z-scored), means of other WMI measures (MD_mean_, ISOVF_mean_, ICVF_mean_ and OD_mean_, z-scored) as secondary outcomes, and a linear model as the final equation (Supplementary Method 3) [35]. All these models were adjusted for baseline age, sex, race, SES, household size and LE8 total score. These markers were also allowed to attenuate the relationship between POHP and WMI. The results are displayed as tabular data with heatmap graphics. The top discovered mediators in the POHP-WMI interaction are highlighted based on their function and connection to POHP and neurodegeneration in earlier investigations.

A principal components analysis (PCA) included any of the pre-selected plasma proteins that were statistically significant mediators with a positive or inverse pure indirect effect at type I error of 0.05 and a statistically significant total effect in the same direction. For ease of comprehension, numerous components that were retrieved underwent orthogonal rotation using varimax. When more than one component was extracted, the Kaiser rule (eigenvalue > 1) was used to determine how many components were extracted. Those PCA scores (z-scores) were forecasted using the regression approach and included in another set of 4-way decomposition models to examine the extent of mediation and/or moderation by these components in the overall effect of POHP on each of the five WMI indicators (z-scored). POHP was used as the exposure, and a structural equations model was used to conduct a sensitivity analysis by adding an increasing number of exogenous factors and calculating the total, direct, and indirect effects from these models with possible final outcomes. The same four-way decomposition models used for the global mean WMI metrics were used for a secondary analysis with tract-specific WMI metrics.

The supplemental materials also contain results for significant pure indirect effects (PIE) across the whole proteome (i.e., *k* = 1463 proteins). These results were entered into the OLINK insight pathways browser (https://github.com/baydounm/UKB-paper10-supplementarydata) to determine the most common pathways used by those mediators in relation to each of the five WMI metrics, which was then displayed as a collection of independent and connected pathways.

Other sensitivity analyses were conducted: (i) Structural Equations Models described above were further adjusted for key time lag variables, including fasting time, season and time between baseline assessment and the neuroimaging visit and time from blood draw to processing of samples for proteomics; (ii) The larger set of plasma proteins which was made available end of 2023 (~3000 proteins) was utilized to conduct part of the analysis, including screening for significant and strong association with POHP, and four-way decomposition models for the selected plasma proteins to detect additional consistent mediators; (iii) POHP was tested as a predictor for all WMI mean measures in the largest available sample with complete dMRI neuroimaging data, including among participants who were excluded due to non-available plasma proteins.

## Results

Table 1 displays the distributions of those key variables overall and stratified by sex. POHP was more prevalent among male (12.3%) compared with female adults (9.6%) in this sample (*P* < 0.001). Sex differences were also notable with an average higher SES z-score among male adults, in contrast with LE8 total and component scores which indicated better cardiovascular health among female adults. Female adults were younger and had on average a smaller household size compared to their male counterparts. Importantly, there were sex differences in both mean MD and mean ISOVF, indicating better brain white matter microstructural integrity (WMI) among female compared to male adults (*P* < 0.001), with no difference detected for FA, ICVF or OD.

**Table 1:** Study sample characteristics by sex: UK biobank 2006–2021.

	Overall (*N* = 5089)	Men (*N* = 2368)	Women (*N* = 2721)	*P*_sex_
Demographic, means ± SE or %				
Baseline age, y	54.57 ± 0.11	55.3 ± 0.16	53.9 ± 0.14	<0.001
Sex, % female	53.4%	0.0	100.0	
Race/ethnicity				
White	96.8%	96.7%	96.9%	(Ref)
Black	0.8%	0.9%	0.7%	0.45
South Asian	0.8%	1.0%	0.6%	0.12
Other	1.6%	1.4%	1.8%	0.37
Non-White, %	3.2	3.3	3.1	0.62
Household size	2.605 ± 0.016	2.670 ± 0.024	2.549 ± 0.023	<0.001
Socioeconomic, means ± SE or %				
*Education*				
Low	15.3%	16.3%	14.5%	0.89
Intermediate	34.8%	31.0%	38.1%	<0.001
High	49.9%	52.7%	47.4%	(Ref)
*Income*				
Less than £18,000	11.1%	8.6%	13.3%	<0.001
£18,000–£29,999	22.0%	20.4%	23.4%	0.039
£30,000–£51,999	30.0%	30.5%	29.6%	(Ref)
£52,000–£100,000	29.0%	31.4%	26.7%	0.079
greater than £100,000	8.0%	9.1%	7.0%	0.043
Townsend Deprivation Index	−1.754 ± 0.040	−1.794 ± 0.058	−1.718 ± 0.054	0.34
SES	−0.010 ± 0.009	+0.033 ± 0.014	−0.047 ± 0.013	<0.001
Poor oral health problems, POHP, %	10.9	12.3	9.6	0.002
Life’s essential 8, means ± SE				
Total score	533.1 ± 1.3	515.8 ± 1.8	548.1 ± 1.8	<0.001
Lifestyle score	262.3 ± 0.8	257.5 ± 1.2	266.5 ± 1.1	<0.001
Biological score	271.3 ± 0.9	258.5 ± 1.3	282.4 ± 1.3	<0.001
Brain dMRI metrics, means ± SE				
Mean FA	+0.5616 ± 0.0003	+0.5617 ± 0.0004	+0.5616 ± 0.0004	0.90
Mean MD	+0.00079 ± 4.67e-07	+0.00080 ± 6.97e-07	+0.00080 ± 6.29e-07	<0.001
Mean ICVF	+0.6121 ± 0.0005	+0.6117 ± 0.0006	+0.6124 ± 0.0005	0.39
Mean ISOVF	+0.0940 ± 0.0002	+0.0952 ± 0.0003	+0.0930 ± 0.0003	<0.001
Mean OD	+0.1276 ± 0.0001	+0.1277 ± 0.0002	+0.1275 ± 0.0002	0.56

No multiple imputation was carried out in this analysis. *P*-value is associated with the parameter for sex in bivariate linear and multinomial logistic regression analyses, with the main outcome being a continuous or categorical characteristic, respectively. (Ref) is the referent category in the multinomial logistic regression model. Values are means ± SE or percentages.

*FA* Fractional Anisotropy, *ICVF* Intracellular Volume Fraction, *ISOVF* Isotropic Volume Fraction, *LE8* Life’s Essential 8, *MD* Mean Diffusivity, *OD* Orientation Dispersion, *Pd* Periodontal Disease, *POHP* Poor Oral Health Problems, *SES* Socio-economic Status, *UK* United Kingdom.

Table 2 shows the overall and sex-specific association between POHP and brain WMI, after adjusting for key potentially confounding covariates. POHP was associated with most metrics of brain WMI, with the exposed group having lower WMI compared to the unexposed. In the overall sample, this pertained to FA_mean_, MD_mean_ and ISOVF_mean_. Interestingly, the positive association between POHP and ISOVF_mean_ was only detected among men, for whom no other POHP-WMI metric association was found. In contrast, among women, POHP was associated with poorer WMI only in terms of lower FA_mean_ and reduced ICVF_mean_ (β < 0, *P* < 0.05). A sensitivity analysis carried out including all available data – i.e. with or without plasma proteomics data -- found similar results which are detailed in Supplementary Table 4. One notable difference was no detectable difference in mean ICVF_mean_ by POHP among women, while overall, POHP was associated with lower ICVF_mean_.

**Table 2:** Poor oral health problems (POHP) and brain white matter microstructural integrity probed using FA, MD, ICVF, ISOVF and OD MRI parameters (mean values across tracts), overall and by sex: OLS multiple linear regression models; UK biobank 2006–2021^a,b^.

	X = POHP, yes vs. no	*P*_POHP_
	β ± SE	
Overall, *N* = 5089		
Y = FA_mean_	−0.0027 ± 0.00089	0.002
Y = MD_mean_	+3.21e−06 ± 1.37e−06	0.019
Y = ICVF_mean_	*−0.0021* ± *0.0013*	*0.092*
Y = ISOVF_mean_	+0.0013 ± 0.0006	0.019
Y = OD_mean_	+0.0006 ± 0.0004	0.14
Men, *N* = 2368		
Y = FA_mean_	−0.0014 ± 0.0012	0.25
Y = MD_mean_	2.87e−06 ± 1.93e−06	0.13
Y = ICVF_mean_	−0.00022 ± 0.00180	0.91
Y = ISOVF_mean_	+0.0020 ± 0.0008	0.008
Y = OD_mean_	+0.0003 ± 0.0007	0.65
Women, *N* = 2721		
Y = FA_mean_	−0.0041 ± 0.0012	0.001
Y = MD_mean_	*3.60e−06* ± *1.97e−06*	*0.067*
Y = ICVF_mean_	−0.0043 ± 0.0018	0.017
Y = ISOVF_mean_	+0.00051 ± 0.0008	0.52
Y = OD_mean_	*+0.0009* ± *0.0005*	*0.077*

*FA* Fractional Anisotropy, *ICVF* Intracellular Volume Fraction, *ISOVF* Isotropic Volume Fraction, *LE8* Life’s Essential 8, *MD* Mean Diffusivity, *OD* Orientation Dispersion, *OLS* Ordinary Least Square, *Pd* Periodontal Disease, *POHP* Poor Oral Health Problems, *UK* United Kingdom.

^a^All linear regression models were adjusted for baseline age, sex, race/ethnicity, household size, SES z-score and LE8 total score.

^b^*P* for null hypothesis that *β* = 0, *z*-test from linear regression model.

**Fig. 2:**
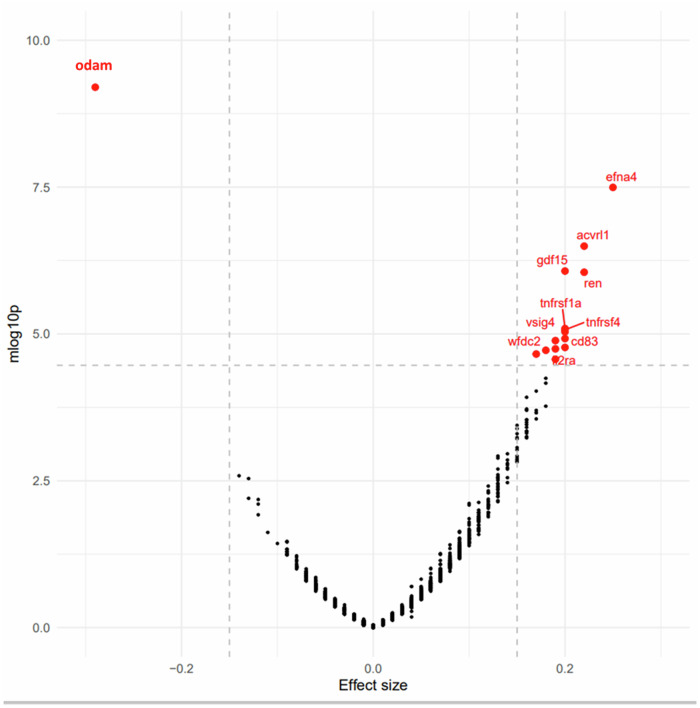
Volcano plot of plasma proteomic biomarkers in relation to poor oral health problems (POHP): UK Biobank 2006–2010. See list of abbreviations for protein abbreviations; LE8 Life’s Essential 8, POHP Poor Oral Health Problems, UK United Kingdom. Note: Based on a series of multiple linear regression models, with main predictor being POHP (1 = yes, 0 = no) and the outcome being each of 1463 plasma proteomic biomarkers (Log2 transformed, z-scored). Models were adjusted for baseline age, sex, race/ethnicity, household size, SES z-score and LE8 total score. The y-axis is the predictor’s associated *p*-value on a -Log10 scale and the X-axis is the β coefficient (effect of POHP (yes vs. no) on standardized z-scores of plasma proteomic markers) from the multiple linear regression models. An estimate with a Bonferroni corrected *p*-value < 0.05 and a lower confidence limit for the 95% CI of effect size >0.15 in absolute value is marked by the plasma proteomic marker abbreviation (See UKB showcase URL: https://biobank.ndph.ox.ac.uk/showcase/). Selected proteins (k = 15) for further mediation analysis have a corrected *p*-value < 0.05 and a point estimate >0.15 in absolute value (red). All other points are shown in blue (corrected *p*-value < 0.05 but effect size < 0 but >−0.15), in orange (corrected *p* < 0.05 but effect size > 0 but <0.15), and in black (corrected *p*-value > 0.05). Details are provided on github: https://github.com/baydounm/UKB-paper10-supplementarydata.

We evaluated 1463 multiple linear regression models to examine the strongest relationships between POHP and the plasma proteome. Only 15 plasma proteins were significantly predicted by POHP after Bonferroni correction, all of which passed the effect size restriction of a beta that was either <−0.15 or >+0.15, reflecting ~1/7-1/6 SD difference between POHP^+^ and POHP^-^ groups in Log2 transformed and z-scored proteins. The key findings are shown as a volcano plot (Fig. 2). Selected strongly associated proteins in their standardized z-scored form with POHP (k = 15) were included into a four-way decomposition models with alternative outcomes being the 5 dMRI metrics, FA_mean_, MD_mean_, ICVF_mean_, ISOVF_mean_ and OD_mean_ (all z-scored) and all equations being multivariable-adjusted linear regression models. As in the previous analyses, potentially confounding covariates were baseline age, sex, race, SES, household size and LE8 total score.

**Fig. 3:**
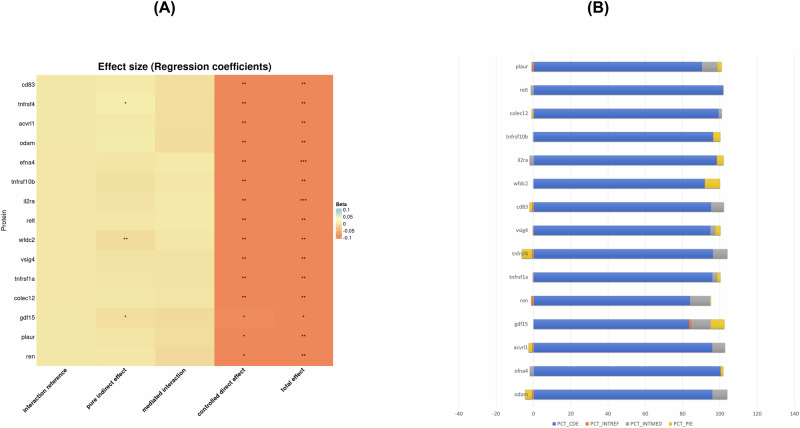
Four-way decomposition of the association between Poor Oral Health Problems (POHP) and the brain white matter microstructural integrity metric, fractional anisotropy (FA, z-scored) by the selected plasma proteomic biomarkers (*k* = 15): UK biobank 2006–2021. **A** Heatmap for raw 4-way decomposition. Note: Effects could range from 0 to 0.5. *P < 0.05; **P < 0.010;***P < *0.001*. **B** Percentages from 4-way decomposition (% of total effect). Note: Proteins are ordered in ascending order of *p*-value for the effect of POHP on the protein after correction for multiple testing and extraction of 18 proteins based on effect sizes <−0.15 or >0.15. PCT_CDE (blue): percent of total effect that is a controlled direct effect; PCT_INTREF (orange): percent of total effect that is interaction referent; PCT_INTMED (gray): percent of total effect that is mediated interaction; PCT_PIE (yellow): percent of total effect that is pure indirect effect. Models were adjusted for baseline age, sex, race/ethnicity, household size, SES z-score and LE8 total score. ereri_cde excess relative risk due to neither mediation nor interaction or controlled direct effect, ereri_intmed excess relative risk due to mediated interaction or mediated interaction, ereri_intref excess relative risk due to interaction only or interaction referent, ereri_pie excess relative risk due to mediation only or pure indirect effect, pct_cde percent of total effect that is controlled direct effect, pct_intmed percent of total effect that is mediated interaction, pct_intref percent of total effect that is interaction referent, pct_pie percent of total effect that is pure indirect effect, Pd Periodontal Disease, POHP Poor Oral Health Problems, tereri Total excess relative risk, UK United Kingdom. See supplementary Table 5 for protein abbreviations. Other Protein abbreviations are found at https://www.ncbi.nlm.nih.gov/gene/.

Table 3, Fig. 3 (both focused on FA_mean_) and supplementary datasheet 1 (all five WMI metrics) present key findings and indicate two of the strongest plasma proteome mediators were growth differentiation factor 15 (GDF15) and WAP four-disulfide core domain 2 (WFDC2; also known human epididymis protein 4; HE4), which consistently and partially mediating the association between POHP and at least 2 of the five WMI metrics. In contrast, in the case of FA_mean_ (Table 3 and Fig. 3), 12 of the 15 proteins did not act as mediators, while one had a mediating effect that was in the opposite direction to the total effect (TNFRSF4), indicating that for the most part, POHP had a direct inverse effect on FA_mean_ that was not explained by the strongest proteomic predictors of this exposure. The 2 mediators of 15 (i.e. GDF15 and WFDC2) which showed a consistently significant pure indirect effect along with a total effect of POHP with *P* < 0.05, are shown in supplementary Table 5 with descriptions of their main biological functions and links to POHP and neurodegeneration based on previous studies. GDF15 and WFDC2, mediated about 7–8% of total effect of POHP on FA_mean_ (PIE < 0, TE < 0, *P* < 0.05) (Table 3 and Fig. 3). Other WMI metrics whereby GDF15 acted as a potential mediator of the main association of interest included MD_mean_ and ISOVF_mean_, whereas this was the case for WFDC2 for MD_mean_ and ICVF_mean_. In both cases (i.e. for GDF-15 and WFDC2), TE was also detected and in the same direction as PIE (Supplementary Datasheet 1). Most notably, GDF15 explained around 13% of the POHP-ISOVF_mean_ association based on the PIE as percent of TE, while WFDC2 explained over 15% of the POHP-ICVF_mean_ association. This percentage was 8–9% in the case of MD_mean_ for those two key proteomic mediators. A sensitivity analysis was conducted using up to 2920 plasma protein made available in November of 2023 to our UKB project. The same exercise was carried out whereby the strongest associations between POHP and each protein were screened for using Bonferroni correction and an absolute value for the effect size set to 0.15, while adjusting for potentially confounding covariates. This resulted in only 12 proteins being selected. Most of the previously selected 15 proteins were retained, including GDF15 and WFDC2. None of the newly selected proteins (RNASE6 and TNFRSF1A) were found to be significant mediators based on a statistically significant PIE at a type I error of 0.05. It is worth noting that the new batch of proteins had markedly smaller sample sizes compared to the older batch, resulting in a reduced statistical power to detect an association with POHP or mediation between POHP and each WMI metric. The full Output is provided in: https://github.com/baydounm/UKB-paper10-supplementarydata.

**Table 3:** Four-way decomposition of the association between Poor Oral Health Problems (POHP) and brain white matter microstructural integrity (Mean fractional anisotropy across tracts, FA_mean_, z-scored) through selected plasma proteomic biomarkers (*k* = 15; *N*_max_ = 5089): UK biobank 2006–2021.

FOURWAYDECOMP	Beta	SE	z	P	LCL	UCL	PROTEIN
te	−0.1378	0.04379	−3.15	0.002	−0.2236	−0.0519	odam
cde	−0.1329	0.04467	−2.98	0.003	−0.2205	−0.0454	odam
intref	0.00116	0.00126	0.92	0.355	−0.0013	0.00364	odam
intmed	−0.01081	0.01063	−1.02	0.309	−0.0316	0.01001	odam
pie	0.00485	0.00431	1.13	0.26	−0.0036	0.0133	odam
pct_cde	96.5						odam
pct_intref	−0.85						odam
pct_intmed	7.85						odam
pct_pie	−3.52						odam
te	−0.1420	0.0439	−3.24	0.001	−0.2280	−0.0560	efna4
cde	−0.1425	0.0453	−3.15	0.002	−0.2313	−0.0537	efna4
intref	−0.0003	0.0010	−0.29	0.773	−0.0022	0.0017	efna4
intmed	0.0027	0.0092	0.29	0.771	−0.0153	0.0207	efna4
pie	−0.0019	0.0037	−0.50	0.616	−0.0092	0.0055	efna4
pct_cde	100.36						efna4
pct_intref	0.21						efna4
pct_intmed	−1.88						efna4
pct_pie	1.32						efna4
te	−0.1330	0.0442	−3.01	0.003	−0.2196	−0.0464	acvrl1
cde	−0.1275	0.0458	−2.78	0.005	−0.2174	−0.0377	acvrl1
intref	0.0010	0.0011	0.88	0.378	−0.0012	0.0031	acvrl1
intmed	−0.0090	0.0089	−1.01	0.315	−0.0265	0.0085	acvrl1
pie	0.0025	0.0034	0.74	0.462	−0.0042	0.0092	acvrl1
pct_cde	95.87						acvrl1
pct_intref	−0.73						acvrl1
pct_intmed	6.75						acvrl1
pct_pie	−1.89						acvrl1
te	−0.1137	0.0445	−2.56	0.011	−0.2010	−0.0265	gdf15
cde	−0.0949	0.0466	−2.04	0.042	−0.1863	−0.0036	gdf15
intref	0.0013	0.0012	1.08	0.28	−0.0011	0.0036	gdf15
intmed	−0.0118	0.0087	−1.36	0.175	−0.0288	0.0052	gdf15
pie	−0.0083	0.0035	−2.35	0.019	−0.0152	−0.0014	gdf15
pct_cde	83.49						gdf15
pct_intref	−1.13						gdf15
pct_intmed	10.36						gdf15
pct_pie	7.29						gdf15
te	−0.1245	0.0431	−2.89	0.004	−0.2090	−0.0400	ren
cde	−0.1121	0.0438	−2.56	0.011	−0.1979	−0.0262	ren
intref	0.0015	0.0012	1.18	0.236	−0.0009	0.0039	ren
intmed	−0.0132	0.0083	−1.59	0.112	−0.0296	0.0031	ren
pie	−0.0007	0.0032	−0.21	0.832	−0.0070	0.0057	ren
pct_cde	90.00						ren
pct_intref	−1.17						ren
pct_intmed	10.62						ren
pct_pie	0.55						ren
te	−0.1227	0.0436	−2.81	0.005	−0.2081	−0.0372	tnfrsf1a
cde	−0.1178	0.0448	−2.63	0.009	−0.2056	−0.0299	tnfrsf1a
intref	0.0004	0.0009	0.42	0.673	−0.0013	0.0020	tnfrsf1a
intmed	−0.0033	0.0075	−0.44	0.662	−0.0180	0.0115	tnfrsf1a
pie	−0.0020	0.0030	−0.66	0.507	−0.0078	0.0038	tnfrsf1a
pct_cde	96.01						tnfrsf1a
pct_intref	−0.29						tnfrsf1a
pct_intmed	2.68						tnfrsf1a
pct_pie	1.60						tnfrsf1a
te	−0.1290	0.0431	−3	0.003	−0.2134	−0.0446	tnfrsf4
cde	*−0.1268*	*0.0438*	*−2.9*	*0.004*	*−0.2126*	*−0.0410*	tnfrsf4
intref	0.0011	0.0010	1.04	0.297	−0.0009	0.0031	tnfrsf4
intmed	−0.0099	0.0072	−1.37	0.17	−0.0240	0.0042	tnfrsf4
pie	0.0066	0.0033	1.99	0.046	0.0001	0.0131	tnfrsf4
pct_cde	98.32						tnfrsf4
pct_intref	−0.84						tnfrsf4
pct_intmed	7.66						tnfrsf4
pct_pie	−5.14						tnfrsf4
te	−0.1231	0.0436	−2.82	0.005	−0.2085	−0.0376	vsig4
cde	−0.1168	0.0448	−2.61	0.009	−0.2046	−0.0291	vsig4
intref	0.0004	0.0009	0.43	0.665	−0.0013	0.0020	vsig4
intmed	−0.0034	0.0075	−0.45	0.653	−0.0181	0.0114	vsig4
pie	−0.0032	0.0030	−1.08	0.281	−0.0091	0.0027	vsig4
pct_cde	94.92						vsig4
pct_intref	−0.30						vsig4
pct_intmed	2.75						vsig4
pct_pie	2.63						vsig4
te	−0.1302	0.0435	−2.99	0.003	−0.2155	−0.0450	cd83
cde	−0.1241	0.0443	−2.8	0.005	−0.2110	−0.0373	cd83
intref	0.0010	0.0010	0.93	0.352	−0.0011	0.0030	cd83
intmed	−0.0088	0.0077	−1.15	0.249	−0.0238	0.0062	cd83
pie	0.0018	0.0029	0.6	0.547	−0.0040	0.0075	cd83
pct_cde	95.32						cd83
pct_intref	−0.74						cd83
pct_intmed	6.77						cd83
pct_pie	−1.35						cd83
te	−0.1325	0.0454	−2.92	0.004	−0.2215	−0.0436	wfdc2
cde	−0.1218	0.0466	−2.61	0.009	−0.2132	−0.0304	wfdc2
intref	0.0000	0.0008	0.02	0.987	−0.0015	0.0016	wfdc2
intmed	−0.0001	0.0073	−0.02	0.987	−0.0144	0.0141	wfdc2
pie	−0.0106	0.0039	−2.7	0.007	−0.0183	−0.0029	wfdc2
pct_cde	91.90						wfdc2
pct_intref	−0.01						wfdc2
pct_intmed	0.09						wfdc2
pct_pie	8.02						wfdc2
te	−0.1386	0.0430	−3.23	0.001	−0.2229	−0.0544	il2ra
cde	−0.1363	0.0437	−3.12	0.002	−0.2221	−0.0506	il2ra
intref	−0.0003	0.0008	−0.38	0.706	−0.0019	0.0013	il2ra
intmed	0.0027	0.0070	0.39	0.697	−0.0110	0.0165	il2ra
pie	−0.0047	0.0031	−1.55	0.121	−0.0107	0.0013	il2ra
pct_cde	98.34						il2ra
pct_intref	0.22						il2ra
pct_intmed	−1.98						il2ra
pct_pie	3.42						il2ra
te	−0.1387	0.0441	−3.15	0.002	−0.2250	−0.0524	tnfrsf10b
cde	−0.1337	0.0452	−2.96	0.003	−0.2224	−0.0451	tnfrsf10b
intref	0.0000	0.0008	−0.05	0.96	−0.0015	0.0015	tnfrsf10b
intmed	0.0004	0.0071	0.05	0.96	−0.0136	0.0143	tnfrsf10b
pie	*−0.0053*	*0.0031*	*−1.69*	*0.091*	*−0.0114*	*0.0008*	tnfrsf10b
pct_cde	96.42						tnfrsf10b
pct_intref	0.03						tnfrsf10b
pct_intmed	−0.26						tnfrsf10b
pct_pie	*3.81*						tnfrsf10b
te	−0.1238	0.0442	−2.8	0.005	−0.2103	−0.0372	colec12
cde	−0.1229	0.0457	−2.69	0.007	−0.2124	−0.0334	colec12
intref	0.0002	0.0008	0.27	0.784	−0.0014	0.0018	colec12
intmed	−0.0020	0.0073	−0.28	0.78	−0.0164	0.0123	colec12
pie	0.0010	0.0028	0.35	0.729	−0.0045	0.0065	colec12
pct_cde	99.31						colec12
pct_intref	−0.18						colec12
pct_intmed	1.65						colec12
pct_pie	−0.78						colec12
te	−0.1272	0.0435	−2.93	0.003	−0.2124	−0.0420	relt
cde	*−0.1293*	*0.0445*	*−2.91*	*0.004*	*−0.2165*	*−0.0422*	relt
intref	−0.0002	0.0007	−0.31	0.755	−0.0017	0.0012	relt
intmed	0.0021	0.0067	0.32	0.75	−0.0110	0.0152	relt
pie	0.0002	0.0028	0.09	0.93	−0.0052	0.0057	relt
pct_cde	101.69						relt
pct_intref	0.18						relt
pct_intmed	−1.68						relt
pct_pie	−0.19						relt
te	−0.1122	0.0437	−2.57	0.01	−0.1977	−0.0266	plaur
cde	−0.1014	0.0447	−2.27	0.023	−0.1891	−0.0138	plaur
intref	0.0010	0.0011	0.93	0.352	−0.0011	0.0032	plaur
intmed	−0.0094	0.0080	−1.18	0.237	−0.0250	0.0062	plaur
pie	−0.0023	0.0028	−0.84	0.403	−0.0078	0.0031	plaur
pct_cde	90.44						plaur
pct_intref	−0.91						plaur
pct_intmed	8.39						plaur
pct_pie	2.08						plaur

Tereri and ereri_cde are interpreted as difference in FA z-score between POHP+ and POHP- groups. Models were adjusted for baseline age, sex, race/ethnicity, household size, SES z-score and LE8 total score. Names of the genes/proteins can be found at: https://www.ncbi.nlm.nih.gov/gene.

*ereri_cde* excess relative risk due to neither mediation nor interaction or controlled direct effect, *ereri_intmed* excess relative risk due to mediated interaction or mediated interaction, *ereri_intref* excess relative risk due to interaction only or interaction referent, *ereri_pie* excess relative risk due to mediation only or pure indirect effect, *FA* Fractional Anisotropy, *LE8* Life’s Essential 8, *pct_cde* percent of total effect that is controlled direct effect, *pct_intmed* percent of total effect that is mediated interaction, *pct_intref* percent of total effect that is interaction referent, *pct_pie* percent of total effect that is pure indirect effect, *Pd* Periodontal Disease, *POHP* Poor Oral Health Problems, *tereri* Total excess relative risk, *UK* United Kingdom.

**Fig. 4:**
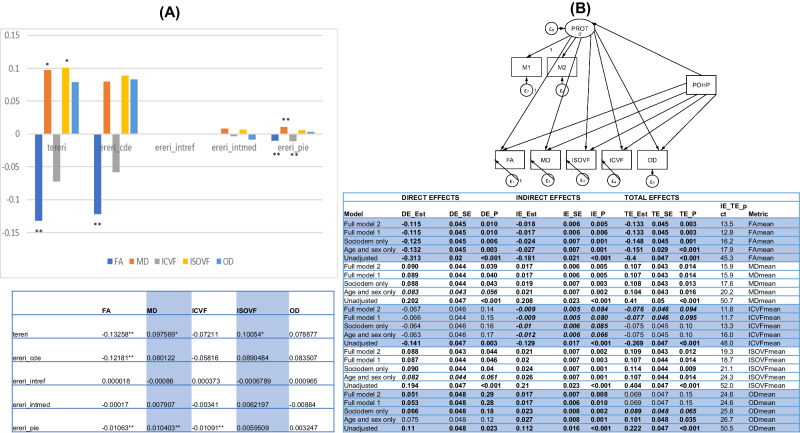
Four-way decomposition and structural equations modeling of the association between Poor Oral Health Problems (POHP) and brain white matter microstructural integrity metrics (FA, MD, ICVF, ISOVF, OD, z-scored) by the single principal component of selected plasma proteomic biomarkers (*k* = 2: GDF15 and WFDC2), overall (*N* = 4663): UK Biobank 2006–2021. **A** Four-way decomposition. **B** Structural Equations Model. DE_EST Direct effect estimate, DE_P Direct effect *p* value, DE_SE Direct effect standard error, ereri_cde excess relative risk due to neither mediation nor interaction or controlled direct effect, ereri_intmed excess relative risk due to mediated interaction or mediated interaction, ereri_intref excess relative risk due, to interaction only or interaction referent, ereri_pie excess relative risk due to mediation only or pure indirect effect, FA fractional anisotropy, ICVF Intracellular Volume Fraction, IE_EST Indirect effect estimate, IE_P Indirect effect *p* value, IE_SE Indirect effect standard error, ISOVF Isotropic Volume Fraction, MD mean diffusivity, OD Orientation Diffusion, Pd Periodontal disease, POHP Poor Oral Health Problems, pct_cde percent of total effect that is controlled direct effect, pct_intmed percent of total effect that is mediated interaction, pct_intref percent of total effect that is interaction referent, pct_pie percent of total effect that is pure indirect effect, TE_EST Total effect estimate, TE_P Total effect *p* value, TE_SE Total effect standard error, tereri Total excess relative risk, UK United Kingdom. See supplementary Table 5 for protein abbreviations. *Note*: Full model 1 included age, sex, race (Non-White vs. White), SES, household size, LE8 total score as exogenous variables; Full model 2 is Full model 1 with additional exogenous variables being fasting time (hours), season at baseline assessment, and time between baseline assessment and neuroimaging visit (days). *P < 0.05; **P < 0.010; ***P < 0.001.

A single PCA factor was extracted from those two key significant proteomic mediators (GDF15 and WFDC2) which was then entered into another series of four-way decomposition and structural equations models (as a sensitivity analysis). This PCA factor explained part of a statistically significant total effect of POHP in the case of FA_mean_ and MD_mean_ only, as depicted in Fig. 4, Panel A. A similar finding was observed in a series of SEM models (Supplementary Datasheet 2 and Fig. 4), that were incrementally adjusted for different sets of exogenous variables. The full model included all the exogenous variables that were entered in four-way decomposition models. Similar to our main models, SEM models indicated that the total effects of POHP on FA_mean_, MD_mean_ and ISOVF_mean_ were statistically significant indicating a deleterious effect of POHP on WMI. Nevertheless, in these models, partial mediation by the PCA score encompassing GDF15 and WFDC2, was observed for all 3 WMI metrics, with percent mediated ranging between 13% for FA_mean_ and 19% for ISOVF_mean_ (Fig. 4, Panel B, Full Model 1). Inclusion of fasting time (hours), season when baseline assessment was carried out and time from baseline assessment to the neuroimaging visit (days) as additional exogenous covariates to the fully adjusted model did not alter findings significantly (Fig. 4, Panel B, Full Model 2).

**Fig. 5:**
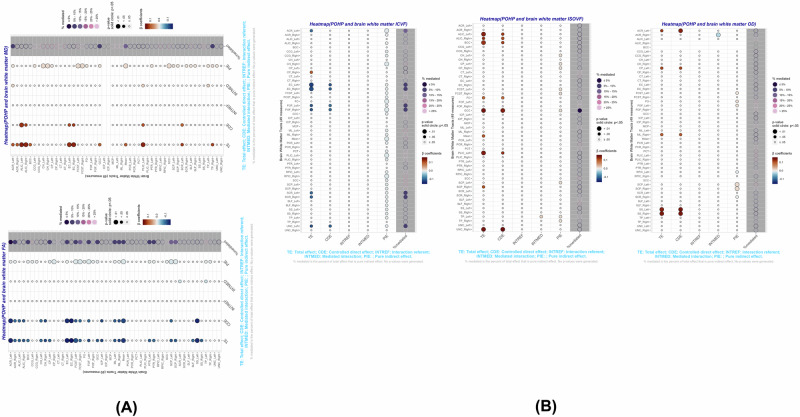
Four-way decomposition of the association between Poor Oral Health Problems (POHP) and brain white matter microstructural integrity metrics (Tract-specific FA, MD, ICVF, ISOVF, OD, z-scored) by the single principal component of selected plasma proteomic biomarkers (*k* = 2: GDF15 and WFDC2), overall (*N* = 4663): UK Biobank 2006–2021. **A** DTI metrics; **B** NODDI metrics. ereri_cde excess relative risk due to neither mediation nor interaction or controlled direct effect, ereri_intmed excess relative risk due to mediated interaction or mediated interaction, ereri_intref excess relative risk due to interaction only or interaction referent, ereri_pie excess relative risk due to mediation only or pure indirect effect, pct_cde percent of total effect that is controlled direct effect, pct_intmed percent of total effect that is mediated interaction, pct_intref percent of total effect that is interaction referent, pct_pie percent of total effect that is pure indirect effect, POHP Poor Oral Health Problems, tereri (or total excess relative risk), UK United Kingdom. See supplementary Table 5 for protein abbreviations. Models were adjusted for baseline age, sex, race/ethnicity, household size, SES z-score and LE8 total score. Other Protein abbreviations are found at https://www.ncbi.nlm.nih.gov/gene/.

Tract-specific results were also explored as a secondary analysis with the common PCA mediator (GDF15 and WFDC2) between Pd and all five WMI metrics. Results are presented in Supplementary Datasheet 3 and visualized in Fig. 5. Total effect of Pd on tract-specific FA was noted in 16 of the 49 tract-specific and global mean metric investigated, with partial mediation seen for the anterior corona radiata (ACR, L/R), body of the corpus callosum (BCC), the cerebellar peduncle (CP, L/R), external capsule (EC, L), fornix cres+stria terminalis (FCST, L/R), the genu of corpus callosum (GCC), the medial lemniscus (L/R), posterior thalamic radiation (PTR, L/R), superior cerebellar peduncle (SCP, L/R), and the Left fronto-occipital fasciculus (FOF, L), all exhibiting negative associations. Several of these tract-specific associations were also seen for other WMI metrics, including MD and ISOVF with positive associations, as expected (Fig. 5 and Supplementary Datasheet 3).

**Fig. 6:**
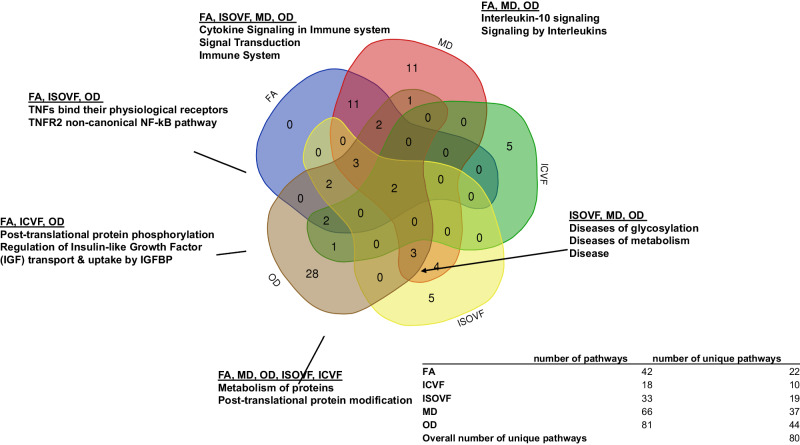
OLINK Insight Pathway browser findings for all plasma proteomic biomarkers found to be significant mediators between periodontal disease and dMRI phenotypes (PIE, *p* < 0.05), (*k* = variable number of proteins per phenotype): UK Biobank 2006–2021. *Source*: https://insight.olink.com/ and http://bioinformatics.psb.ugent.be/webtools/Venn/. The full list of plasma protein biomarkers included into the insight pathway analysis per phenotype is found on github: https://github.com/baydounm/UKB-paper10-supplementarydata. PIE Pure indirect effect. Protein abbreviations are found at https://www.ncbi.nlm.nih.gov/gene/.

We performed OLINK insight pathway analysis including all statistically significant mediators with PIE < 0.05 (*k* = 6 proteins for FA_mean_, *k* = 7 for MD_mean_, *k* = 3 for ICVF_mean_, *k* = 3 for ISOVF_mean_, and *k* = 5 for OD), irrespective of whether those were initially selected using Bonferroni correction. Pathways that were in common to all five phenotypes included metabolism of proteins, while the immune system and signal transduction appeared to be involved in the pathway between POHP and WMI for 4 out of 5 metrics. Detailed pathways for each phenotype are presented in Supplementary Datasheet 4 and visualized in Fig. 6. All supplementary datasheets, detailed code and related result datasets used to generate the Figures and Tables are provided in: https://github.com/baydounm/UKB-paper10-supplementarydata.

Finally, a sensitivity analysis indicated that FA_mean_ was reduced for 3 of the 4 alternative exposures defined earlier, excluding the poor oral health self-reported exposure that combined both severe and non-severe conditions. In contrast, the PCA score for GDF15 and WFDC2 was only significantly increased in one alterative exposure of four, namely the severe self-reported oral problems, 0 = None, 1 = either dentures or loose teeth and 2 = Both. This sensitivity analysis is made available on github at : https://github.com/baydounm/UKB-paper10-supplementarydata.

## Discussion

We used four-way decomposition models to evaluate the total POHP effect on selected MRI-based brain measures through plasma proteomic biomarkers among a sample of 5089 UK Biobank participants aged 40–70 years. We observed an inverse association between POHP and WMI on most metrics, in a sex-specific manner. Of 15 plasma proteins strongly associated with POHP, GDF15 and WFDC2 were consistent mediators. Both plasma proteins mediated about 7–8% of total effect of POHP on FA_mean_ (PIE < 0, TE < 0, *P* < 0.05). SEM yielded significant total effects for FA_mean_, MD_mean_ and ISOVF_mean_ in full models, with percent mediated by a common latent factor for GDF15 and WFDC2 ranging between 13% for FA_mean_ and 19% for ISOVF_mean_. Significant mediation by this common factor was mostly found for 16 out of 49 tract-specific and global mean metrics, in the case of FA. Metabolism of proteins, immune system and signal transduction were the most common pathways involved in these mediational effects.

Recent studies have shown that POHP or specifically Pd, were associated with poorer WMI and other brain MRI markers of the dementia phenome [12, 13]. For instance, a recent study investigated the relationship between the number of teeth present (NTP) and hippocampal atrophy in late middle-aged and older adults. Results suggested that fewer teeth were associated with a faster rate of left hippocampal atrophy in patients with mild periodontitis, while having more teeth was linked to a faster rate of atrophy in those with severe periodontitis [12]. Furthermore, The PAROBRAIN study examined the link between periodontal health, WMI, and cerebral small vessel disease in the Hamburg City Health Study. Results showed that increased white matter hyperintensity was associated with more CAL, plaque index, and tooth loss [13]. Similar to these previous studies, we found that POHP was associated with poorer WMI, using a set of pre-clinical measures that are highly predictive of dementia incidence. This association was found among both men and women, though for different WMI metrics.

The relationships between Pd and dementia are complicated and may include systemic factors, shared risk factors, chronic inflammation, and the presence of bacteria [36–40]. Although these pathways have not been proven, they provide information about a potential connection between these two illnesses. For instance, Pd causes long-lasting gum inflammation and releases cytokines and pro-inflammatory chemicals into the bloodstream [39, 40]. These inflammatory molecules may enter the brain and contribute to neuroinflammation, a characteristic of dementia. Additionally, inflammation may compromise the blood-brain barrier, allowing inflammatory agents and germs from the oral cavity to enter the brain and possibly accelerate cognitive impairment. These bacteria may then cause an immunological reaction that damages and inflames the brain. Studies have even found periodontal bacteria in brain samples from dementia patients, indicating a potential link [36–40]. Another element that might be important is systemic inflammation [39, 40]. A systemic inflammatory condition that is brought on by persistent Pd inflammation has been linked to several chronic illnesses, including dementia [39, 40]. Finally, Pd and dementia risk factors that are similar are probably involved [39, 40]. Age, genetic and biological variables, lifestyle decisions including smoking, and other risk factors are all shared by Pd and dementia [36, 37]. While these proposed routes shed light on the correlations between Pd and dementia that have been identified, more investigation is required to establish causal links and identify the precise processes at work. However, treating Pd may have wider effects on overall health, including brain health, highlighting the need of good oral hygiene as a potential preventive practice for dementia.

Our study suggests that specific circulating proteins as well as several pathways are involved in mediating Pd’s association with poorer WMI. One key protein found to be an important mediator between POHP and WMI was WFDC2. WFDC2, also known as human epididymal protein 4 (HE4), is a secreted glycoprotein that may play a role in periodontal health through extracellular matrix remodeling. A recent study using data from National Health and Nutrition Examination Survey (NHANES) 2001–2002, found that in women (*n* = 1715) over the age of 30 that high serum levels of HE4 were associated with Stage III/IV periodontitis [41]. Serum HE4 levels were associated with cognitive decline in patients with diabetes mellitus [42] and with cognitive decline and dementia in the British Whitehall II and US Atherosclerosis Risk in Communities (ARIC) studies [18]. However, HE4 has not been extensively studied in relation to brain MRI markers of the dementia phenome.

Among the most important mediators was GDF15, which is a stress-response protein that plays a multitude of roles in disease processes, but recently has been linked to the immunological response to infection [34, 43] as well as playing a potential role in cognition and dementia [19, 44, 45]. There is some evidence that GDF15 may also play a role in Pd [46–49]. Emerging evidence has also connected GDF15 to cognition, dementia, and AD [44, 50–54]. In a recent study, GDF15 and NT-proBNP were associated with increased risk of incident all-cause and AD dementia in individuals over 60 years [54]. These biomarkers improved dementia risk classification beyond traditional clinical risk factors, indicating their potential for predicting dementia incidence. The same study also showed that elevated GDF15 was associated with lower total brain and hippocampal volumes, greater white matter hyperintensity volume, and poorer cognitive performance [54]. Another study of older individuals found that mean FA values globally and in various brain regions were negatively associated with GDF15 serum levels. Tract-specific findings were comparable to our present study, particularly the inverse association with FA in the corpus callosum, the posterior thalamic radiation [55]. An earlier study examined the relationship between GDF15 levels and human brain gray matter (GM) volumes in a community-dwelling sample aged 70–90 years [56]. Results showed significant negative associations between GDF15 serum levels and both subcortical and cortical GM volumes [56]. Increases in GDF15 serum levels were associated with decreases in GM volumes over two years [56]. Similarly, in the Framingham Offspring cohort study, higher GDF15 was linked to greater white matter hyperintensity volumes [57].

Our current study has many strengths with notable limitations. First, this study examined a wide-scale proteomic analysis in a large cohort, and it is the first cohort study with sufficient power to assess the plasma proteome’s mediating and moderating effects in the relationship between Pd and measures of brain white matter microstructural integrity. Second, using specific diagnosis dates received through record linkage, UK Biobank investigators generated diagnosed Pd among others. Third, the UK Biobank includes a broad range of topics, allowing for unbiased estimates of exposure-outcome relationships via confounder adjustment. Potential study limitations include selection bias due to missing data and measurement error induced by employing self-report and ICD-10 codes. The definition of the exposure required making a choice between several combinations of both, even though a partial sensitivity analysis was carried out on other key exposure definitions. Although key confounders were accounted for, residual confounding is possible due to the observational nature of this study. Furthermore, while prevalent dementia cases were excluded up to three years after baseline assessment, reverse causality remains a possible explanation for the association between Pd and sub-clinical phases of dementia. Further, while highly sensitive to capturing early damage to cerebral tissue microstructure, DTI indices are not specific to any underlying determinant of the brain tissue, which hampers specific interpretation of the results [58–60]. While the NODDI analysis has been introduced to provide more specificity to neurite degeneration and dispersion through ICVF and OD metrics, derived values using NODDI suffer from various experimental and modeling limitations. Indeed, derived ICVF and ISOVF parameters have been shown to be sensitive to the choice of the echo time and the underlying assumptions of the fixed diffusivity parameters adopted in the NODDI modeling [58–60]. Therefore, further examinations are still required to validate our results. Moreover, four-way decomposition method has assumptions and limitations that are discussed elsewhere [35]. Finally, because the UK Biobank is underpowered for such analyses, our analyses did not stratify by racial/ethnic groupings. Future research should strive to include a stratified analysis across all main racial/ethnic groups using a larger sample of the UK biobank.

## Conclusions

In summary, POHP was linked to lower WMI, which was mediated in part by two major plasma proteins, GDF15 and WFDC2. Future research should focus on other common infection types as well as inflammatory conditions that have previously been shown to predict dementia incidence, determining whether similar mechanisms are involved across types of infections and inflammatory conditions, and assessing the validity of these findings in populations at highest risk for dementia in the UK.

## Supplementary information

Online Supplementary Materials

Datasheet 1

Datasheet 2

Datasheet 3

Datasheet 4

## Data Availability

The data analyzed in this study is subject to the following licenses/restrictions: UK Biobank is a large-scale biomedical database and research resource, containing in-depth genetic and health information from half a million United Kingdom participants. The database is regularly augmented with additional data and is globally accessible to approved researchers undertaking vital research into the most common and life-threatening diseases. Requests to access these datasets should be directed to https://www.ukbiobank.ac.uk/.
