# Supplementary material for: Mediating and moderating effects of plasma proteomic biomarkers on the association between poor oral health problems and brain white matter microstructural integrity: the UK Biobank study
Source: Mol Psychiatry. 2024 Jul 30;30(2):388–401. doi: 10.1038/s41380-024-02678-3 (PMC11746130; doi:10.1038/s41380-024-02678-3)
Supplement: Supplementary file 1 — Online Supplementary Materials [file 41380_2024_2678_MOESM1_ESM.pdf]

ONLINE SUPPLEMENTARY MATERIALS

Mediating and moderating effects of plasma proteomic biomarkers on the association between poor oral health problems and brain white matter microstructural integrity: The UK Biobank study

May A. Beydoun . al

OSM1: Diffusion-weighted MRI: Diffusion-weighted imaging (DWI) acquisition and processing

Main source: http://biobank.ctsu.ox.ac.uk/crystal/docs/brain_mri.pdf)

The diffusion magnetic resonance imaging (*dMRI*) acquisition consisted of a spin-echo echo-planar MRI sequence with 10 T_2-weighted volumes acquired at b = 0 s/mm^2 , 50 volumes at b = 1000 s mm^{-2} and 50 volumes at b = 2000 s/mm^2 , for a total of 100 different diffusion-encoding directions. All images/volumes were acquired with 2 mm isotropic voxel size, multiband acceleration factor of 3, repetition time (TR) of 3600 ms, and echo time (TE) of 92 ms. Detailed processing information for DWI is provided elsewhere [1]. Briefly, the preprocessing includes eddy current distortion correction, head motion correction, and outlier slices correction using the FSL's Eddy tool [2-4].

DTI analysis: the preprocessed DW images (b = 0 s mm^{-2} and b = 1,000 s/mm^2) were used to derive corresponding fractional anisotropy (FA) and mean diffusivity (MD) maps by fitting a diffusion tensor model using the FSL's DTIFIT tool [2, 5]. FA reflects the directionality of molecular displacement by diffusion, with values between 0 and 1; higher values are an indicator of anisotropic diffusion that confers a preferred direction, whereas MD reflects the average magnitude of molecular displacement by diffusion, with higher values indicating more freely diffusing water. In WM, decreased FA or increased MD may suggest deterioration of axons or myelin sheaths, whereas in GM, it may suggest loss of synapses or dendrites. The FA and MD measures were restricted to the white matter skeleton tract. To define the white matter skeleton, parameter maps of FA were registered to the JHU white matter Atlas (ICBM-DTI-81) [2, 6] and, using FSL's Tract-Based Spatial statistics (TBSS) [2, 7], TBSS-derived measures were computed by averaging the skeletonized image of each FA map across subjects within a set of 48 standard-space tract masks.

NODDI analysis: In addition to the DTI fitting, dMRI data was processed using the NODDI analysis (Neurite Orientation Dispersion and Density Imaging) using the AMICO (Accelerated Microstructure Imaging via Convex Optimization) tool https://github.com/daducci/AMICO [8, 9]. NODDI provides measures of the intra-cellular volume fraction (ICVF), an index of white matter axonal density, the isotropic water volume fraction (ISOVF), a measure of the freely-moving water generally attributed to CSF, and the orientation dispersion index (ODI), a measure of axonal disorganization. Similarly, the derived NODDI outcome maps were registered to the JHU atlas and ICVF, ISOVF and ODI were calculated within a set of 48 standard-space tract masks in the skeleton image described above.

OSM 2: Life's essential 8 and the dietary component

In 2010, the American Heart Association (AHA) widened its scope of interest by prioritizing wellness over illness, by defining a new measure of cardiovascular health (CVH) aiming at individual and population-level health promotion[10, 11]. CVH was defined with 7 potentially modifiable biological and lifestyle factors, that when at optimal levels, would result in greater cardiovascular disease (CVD)-free survival, longevity, and better quality of life. This measure of CVH was labelled "Life's Simple 7" (LS7), with its 7 components including indicators of diet quality, greater physical activity, reduced cigarette smoking, lower body mass index, total cholesterol, fasting blood glucose, and optimal blood pressure levels. Using clinical thresholds that were accepted for both children and adults, each metric was categorized as poor (0), intermediate (1), or ideal (2). The overall summary score of LS7 could range from 0 (all metrics at poor levels) to 14 (all 7 metrics at ideal levels)[10, 11]. Since 2010 AHA statement was published, CVH was re-evaluated and an AHA Presidential Advisory proposed an enhanced version of CVH, reflecting advances made over a decade of research, while LS7's methodological limitation have been remedied[11, 12]. This new measure was labeled "Life's Essential 8" (LE8), retaining all 7 components of LS7 with major modifications to definitions and scales (described below). Sleep health was added to generate life's essential 8 (LE8) given its known influence on CVH across the life span[11, 12], (**OSM 2**, **Supplementary Table 1**, for detailed algorithm used to generate LE8 total score).

Supplementary Table 1. Definition and scoring approach for quantifying cardiovascular health, as per the American Heart Association's Life's Essential 8 score [11, 12], and as applied in the National Health and Nutrition Examination Surveys, 2013-2018

Domain	CVH Metric	Method of Measurement	Quantification of CVH Metric - Adults<br><br>(≥20 Years)
**Health Behaviors**	**Diet**	**Measurement: Self-reported daily intake of a DASH-style eating pattern**<br>**Example tools for measurement: DASH diet score (populations)**	Quantiles of DASH-style diet adherence<br>**Scoring(Population):**<br><u>Points</u> <u>Quantile</u><br>100 ≥95th %ile (top/ideal diet)<br>80 75th – 94th %ile<br>50 50th – 74th %ile<br>25 25th – 49th %ile<br>0 1st – 24th %ile (bottom/least ideal quartile)
	**Physical activity**	**Measurement: Self-reported minutes of moderate or vigorous physical activity per week**<br>**Example tools for measurement:**<br>NHANES PAQ-K questionnaire	**Metric: Minutes of moderate (or greater) intensity activity per week**<br>**Scoring:**<br><u>Points</u> <u>Minutes</u><br>100 ≥150<br>90 120 – 149<br>80 90 – 119<br>60 60 – 89<br>40 30 – 59<br>20 1 – 29<br>0 0

	**Nicotine exposure**	**Measurement:** Self-reported use of cigarettes or inhaled nicotine- delivery system<br>**Example tools for measurement:**<br>NHANES SMQ	**Metric: Combustible tobacco use and/or inhaled NDS use; or secondhand smoke exposure**<br>**Scoring:**<br>Points Status<br>100 Never smoker<br>75 Former smoker, quit≥5 yrs<br>50 Former smoker, quit 1 - <5 yrs<br>25 Former smoker, quit <1 year, or currently using inhaled NDS<br>0 Current smoker<br>Subtract 20 points (unless score is 0) for living with active indoor smoker in home
	**Sleep health**	**Measurement:** Self-reported average hours of sleep per night<br>**Example tools for measurement:**<br>"On average, how many hours of sleep do you get per night?" Consider objective sleep/actigraphy data from wearable technology, if available	**Metric:** Average hours of sleep per night<br>**Scoring:**<br>Points Level<br>100 7 – <9<br>90 9 - <10<br>70 6 - <7<br>40 5 -<6 or≥10

			20 4 - <5<br>0 <4
**Health Factors**	**Body mass index**	**Measurement:** Body weight (kg) divided by height squared (m2) **Example tools for measurement:** Objective measurement of height and weight	**Metric :Body mass index (kg/m²)**<br>**Scoring:**<br>Points Level<br>100 <25<br>70 25.0 – 29.9<br>30 30.0 – 34.9<br>15 35.0 – 39.9<br>0≥40.0
	**Blood lipids**	**Measurement:** Plasma total and HDL-cholesterol with calculation of non-HDL-cholesterol<br>**Example tools for measurement:**<br>Fasting or non-fasting blood sample	**Metric :Non-HDL-cholesterol (mg/dL)**<br>**Scoring:**<br>Points Level<br>100 <130<br>60 130 – 159<br>40 160 – 189

			20 190 – 219<br>0 ≥220<br>If drug-treated level, subtract 20 points
	**Blood glucose**	**Measurement: Fasting blood glucose or casual hemoglobin A1c Example tools for measurement: Fasting (FBG, HbA1c) or non-fasting (HbA1c) blood sample**	**Metric: Fasting blood glucose (mg/dL) or Hemoglobin A1c (%)**<br>**Scoring:**<br>Points Level<br>100 No history of diabetes and FBG <100 (or HbA1c < 5.7)<br>60 No diabetes and FBG 100 – 125 (or HbA1c 5.7-6.4) (Pre-diabetes)<br>40 Diabetes with HbA1c <7.0<br>30 Diabetes with HbA1c 7.0 – 7.9<br>20 Diabetes with HbA1c 8.0 – 8.9<br>10 Diabetes with Hb A1c 9.0 – 9.9<br>0 Diabetes with HbA1c ≥10.0

	**Blood pressure**	**Measurement:** Appropriately measured systolic and diastolic blood pressure<br>**Example tools for measurement:** Appropriately sized blood pressure cuff	**Metric:** Systolic and diastolic blood pressure (mm Hg)<br>**Scoring:**<br>Points Level<br>100 <120/<80 (Optimal)<br>75 120-129/<80 (Elevated)<br>50 130-139 or 80-89 (Stage I HTN)<br>25 140-159 or 90-99<br>0 ≥160 or≥100

The touchscreen questionnaire of the UKB main study included twenty-nine questions regarding diet and eighteen questions related to alcohol. The touchscreen questionnaire inquired about food consumption frequency and nature, over the past year of the following food groups: cooked vegetables, salad/raw vegetables, fresh fruit, dried fruit, oily fish, other fish, processed meats, poultry, beef, lamb, pork, cheese, salt added to food, tea, water, as well as questions on the type of milk most commonly consumed, type of spread most commonly consumed, number of slices and type of bread most commonly consumed, number of bowls and type of breakfast cereal most commonly consumed, cups of coffee and type most commonly consumed, as well as questions on the avoidance of specific foods and food groups (eggs, dairy products, wheat, sugar), age last ate meat (for participants who reported never consuming processed meats, poultry, beef, lamb or pork), temperature preference of hot drinks, changes in diet in the past 5 years, and variation in diet. Four of the

dietary questions originally utilized in the pilot trial were slightly altered for the main assessment phase: these were the items related to avoiding specific foods and food groups; spread type; bread type; and variation in diet.

The Healthy Diet Index (HDI) score combined several food groups in terms of quantity and frequency of consumption per week, when available to reflect the guidelines listed in **Supplementary Table 2**. However, those criteria were modified to fit the availability of data in the UK biobank. **Supplementary Table 3** represents the food groups that were selected, their respective coding scheme and the scoring system to reflect better diet quality, approximating the criteria in **Supplementary Table 2**. The touchscreen questionnaire was later validated against the 24-hr recall that was administered over time to UK biobank participants and has shown adequate agreement in terms of ranking for each food group of interest[13].

Supplementary Table 2. Goals and guidelines used to construct the Healthy Diet Score

Consume more	Goal*	One Serving Equals…
**Fruits**	3 servings/d	1 medium-sized fruit; ½ cup of fresh, frozen, or unsweetened canned fruit; ½ cup of dried fruit; ½ cup of 100% juice
**Nuts, seeds**	4 servings/wk	1 ounce
**Vegetables, including legumes (excluding russet or white potatoes)**	3 servings/d	1 cup of raw leafy vegetables; ½ cup of cut-up raw vegetables, cooked vegetables, or 100% vegetable juice
**Whole grains†**	3 servings/d, in place of refined grains	1 slice of whole-grain bread; 1 cup of high-fiber, whole-grain cereal; ½ cup of cooked whole-grain rice, pasta, or cereal

**Fish, shellfish**	≥2 servings/wk	3.5 ounces (100 g)
**Dairy products, especially yogurt and cheese ‡**	2–3 servings/d	1 cup of milk or yogurt; 1 ounce of cheese
**Vegetable oils**	2–6 servings/d	1 teaspoon oil, 1 tablespoon vegetable spread
**Consume less**		
**Refined grains, starches, added sugars†**	No more than 1–2 servings/d	
**Processed meats**	No more than 1 serving/wk	1.75 ounces (50 g)
**Unprocessed red meats**	No more than 1–2 servings/wk	3.5 ounces (100 g)
**Industrial trans fat §**	Don't eat	Any food containing or made with partially hydrogenated vegetable oil
**Sugar-sweetened beverages**	Don't drink	8 ounces of beverage; 1 small sweet, pastry, or dessert
**Sodium**	No more than 2000 mg/d	n/a

Source: https://www.ahajournals.org/doi/10.1161/CIRCULATIONAHA.115.018585#d3e341

* Based on a 2000 kcal/d diet. Servings should be adjusted accordingly for higher or lower energy consumption.
† As a practical rule-of-thumb for selecting healthful whole grains and avoiding carbohydrate-rich products high in starches and added sugars, the ratio of total carbohydrate to dietary fiber (g/serving of each) appears useful. Foods with ratios <10:1 are preferable; ie, food containing at least 1 g of fiber for every 10 g of total carbohydrate. In addition, minimally processed whole grains (eg, steel-cut oats, stone-ground bread) are generally preferable to finely milled whole grains (eg, many commercial whole-grain breads and breakfast cereals) because of the larger glycemic responses of the latter.
‡ Current evidence does not permit clear differentiation of whether low-fat or whole-fat products are superior for cardiometabolic health. Other characteristics, such as probiotic content or fermentation, may be far more relevant than fat content.
§ The US Food and Drug Administration recently ruled that the use of partially hydrogenated vegetable oils is no longer "generally regarded as safe," which should effectively eliminate the majority of industrial trans fats from the US food supply. Several countries including Denmark, Argentina, Austria, Iceland, and Switzerland have effectively eliminated the use of partially hydrogenated vegetable oils through direct legislation on the amounts of allowable trans fats in foods. Small amounts of certain trans fatty acids may be formed through other industrial processes, including oil deodorization and high-temperature cooking; the health effects of these trace industrial trans fats require careful investigation.

Supplementary Table 3. Healthy Diet Index, HDI, using touchscreen questionnaire in the UK biobank study

Food group/nutrient item	UKB fields used	Definition of meeting criterion	Criteria and scoring
**Consume more**			
Fruits, fresh or dried	1309 and 1319	≥3 servings per day including fresh and dried fruits<br><br>1 piece of dried fruit (e.g. apricot)~=2.5 TBSP, 1 TBSP= 0.063 cups; ½ cup of dried fruit (1 serving) is 3 pieces of dried fruit.<br><br>1 medium sized fruit is one serving.	1=meets criterion, 0=does not meet criterion
Vegetables, salad/cooked	1289 and 1299	≥3 servings per day Including salad, raw and cooked<br><br>1 cup of raw leafy vegetables is 16 TBSP. ½ cup of cooked or non-leafy raw vegetables is 8 TBSP.<br><br>1 serving of raw leafy or non-leafy vegetables is on average ~=12 TBSP; 1 serving of cooked vegetables is ~=8 TBSP	1=meets criterion, 0=does not meet criterion
Whole grains		≥3 servings per day	1=meets criterion, 0=does not meet criterion
**Slices of bread**	1438 and 1448	Daily slices of wholemeal or wholegrain bread (servings per day), convert from weekly slices.	
**Cereal**	1458 and 1448	Daily bowls of whole wheat cereal as servings/day (bran	

		cereal, biscuit cereal, oat cereal and muesli), convert from weekly bowls.	
Fish shellfish	1329 and 1339	Sum weekly frequencies to obtain total servings/week. ≥2 servings/wk	1=meets criterion, 0=does not meet criterion
**Oily fish**	…	…	
**Non-oily fish**	…	…	
Dairy products	6114, 1408 and 1418	Reporting consumption of two milk items and eating cheese once a day to meet the 2-3 servings/day criterion.	1=meets criterion, 0=does not meet criterion
**Milk**	…		
**Cheese**	…		
Vegetable oil	2654	Reporting use of olive oil or polyunsaturated/sunflower oil (yes=1, 0=no)	1=meets criterion, 0=does not meet criterion
**Consume less**			
Refined grains, starches, added sugars†	1438 and 1448	Follow a similar coding scheme as for whole grains but select non-whole grains; <1.5 servings per day	1=meets criterion, 0=does not meet criterion
Processed meats	1349	Once a week or less would meet the criterion.	1=meets criterion, 0=does not meet criterion
Unprocessed red meats	1369, 1379, and 1389	Summation of frequency of consumption across three types of red meats (lamb/mutton, beef or pork).<br><br><3 on the summation corresponds to the criterion of <1-2 servings per week.	1=meets criterion, 0=does not meet criterion
Industrial trans fat §	1428	Never use spread, e.g. butter or margarine etc. would meet the criterion	1=meets criterion, 0=does not meet criterion
Sugar-sweetened beverages	6144	Never eat sugar or food/drink containing sugar would meet the criterion	1=meets criterion, 0=does not meet criterion

Sodium	1478	Salt added to food, never or rarely would meet the criterion	1=meets criterion, 0=does not meet criterion

Source: https://biobank.ndph.ox.ac.uk/showcase/label.cgi?id=100052.

Stata code can be made available upon request.

OSM 3: Four-way decomposition and structural equations models

Each plasma proteomic biomarker was entered as a different potential mediator or moderator in the relationship between PD and white matter integrity (WMI) metrics. When a mediator with which the exposure may interact is present, the overall effect of the PD exposure on WMI metrics was broken down into four distinct parts: (i) neither mediation nor interaction; (ii) interaction alone (and not mediation); (iii) both mediation and interaction; and (iv) only mediation (but not interaction). This recently proposed approach in Stata, which enables the estimation of the four-way decomposition using parametric or semi-parametric regression models, integrates methods to assign effects to interactions with methods to test mediation. Importantly, *Med4way* command [14] [https://github.com/anddis/med4way] was used to test mediation and interaction of the total effect of the "periodontal disease (yes vs. no)" exposure on the all-cause dementia outcome, with up to 1,463 plasma proteomic mediators considered as alternative potential mediator/moderator, using OLS for the final equation with the outcome as well as the equation between exposure and each mediator/moderator. Four-way decomposition was applied to the total sample, using as exogenous variables age, sex and race (Non-White vs. White), SES z-score, household size and life's essential 8 total score. Type I error was set at 0.05 for all analyses.

As a sensitivity analysis, structural equations modeling was carried out whereby GDF-15 and WFDC-2 were entered as measured variable into a latent factor model. The latent factor "PROT" was predicted by PD exposure and predicted all five WMI global mean metrics, allowing their residuals to be correlated. "PD"'s direct effect on each of the five WMI outcomes was also included in the model. The direct, indirect and total effects of PD were estimated from fully adjusted models (with all exogenous variables as for the med4way models), and reduced models (only socio-demographics and unadjusted). Results are presented in **supplementary datasheet 2**.

Supplementary Table 4. Poor oral health problems (POHP) and brain white matter microstructural integrity probed using FA, MD, ICVF, ISOVF and OD MRI parameters (mean values across tracts), overall and by sex: OLS multiple linear regression models; UK biobank 2006-2021[a,b]

	**X=POHP, yes vs. no**	**P_{POHP}**
	**β±SE**	
**Overall, N=39,391**		
$Y=FA_{mean}$	**-0.0017±0.00030**	**<0.001**
$Y=MD_{mean}$	**+2.21e-06±4.68e-07**	**<0.001**
$Y=ICVF_{mean}$	**-0.0017±0.0044**	**<0.001**
$Y=ISOVF_{mean}$	**+0.0007±0.0002**	**0.001**
$Y=OD_{mean}$	+0.0002±0.0002	0.26
**Men, N=18,579**		
$Y=FA_{mean}$	**-0.0019±0.0004**	**<0.001**
$Y=MD_{mean}$	**+2.79e-06±6.67e-07**	**<0.001**
$Y=ICVF_{mean}$	**-0.0024±0.0006**	**<0.001**
$Y=ISOVF_{mean}$	**+0.0007±0.0003**	**0.006**
$Y=OD_{mean}$	+0.0000±0.0002	0.87
**Women, N=20,812**		
$Y=FA_{mean}$	**-0.0014±0.0004**	**0.001**

Y=MD_{mean}	**1.52e-06±6.56e-07**	**0.021**
Y=$ICVF_{mean}$	-0.0009±0.0006	0.12
Y=$ISOVF_{mean}$	***+0.00053±0.0003***	***0.054***
Y=OD_{mean}	+0.0003±0.0002	0.17

Abbreviations: FA=Fractional Anisotropy; ICVF=Intracellular Volume Fraction; ISOVF=Isotropic Volume Fraction; LE8=Life's Essential 8; MD=Mean Diffusivity; OD=Orientation Dispersion; OLS=Ordinary Least Square; Pd=Periodontal Disease; POHP=Poor Oral Health Problems; UK=United Kingdom

[a] All linear regression models were adjusted for baseline age, sex, race/ethnicity, household size, SES z-score and LE8 total score.

[b] P for null hypothesis that β=0, *z*-test from linear regression model.

Supplementary Table 5. DESCRIPTION OF TOP HITS FOR PROTEOMIC MEDIATORS BETWEEN PERIDONTAL DISEASE AND DEMENTIA

Symbol	Protein/Gene name	function	Recent papers with relevance to infection including periodontal disease	Recent papers with relevance to dementia
**gdf15**	**Growth differentiation factor 15**	The protein is expressed in a variety of cell types, functions as a pleiotropic cytokine, and participates in the stress response pathway of cells following cellular injury. Protein levels are elevated in disease conditions like tissue hypoxia, inflammation, acute damage, and oxidative stress.	15-27	28-33
wfdc2	WAP four-disulfide core domain 2	This gene produces a protein that belongs to the WFDC domain family. The WFDC domain, also known as the WAP Signature motif, includes eight cysteines that create four disulfide connections at the protein's core and works as a protease inhibitor in several family members. This gene is expressed in pulmonary epithelial cells and has been linked to various ovarian malignancies. The encoded protein is a tiny secretory protein that may have a role in the development of sperm.	34-37	38, 39

Protein abbreviations are found at https://www.ncbi.nlm.nih.gov/gene/.

SUPPLEMENTARY REFERENCES

1. Gossai A, Waterboer T, Nelson HH, Michel A, Willhauck-Fleckenstein M, Farzan SF *et al.* Seroepidemiology of Human Polyomaviruses in a US Population. *American journal of epidemiology* 2016; **183**(1)**:** 61-69.

2. Badji A, Cohen-Adad J, Girouard H. Relationship Between Arterial Stiffness Index, Pulse Pressure, and Magnetic Resonance Imaging Markers of White Matter Integrity: A UK Biobank Study. *Front Aging Neurosci* 2022; **14:** 856782.

3. Andersson JL, Sotiropoulos SN. Non-parametric representation and prediction of single- and multi-shell diffusion-weighted MRI data using Gaussian processes. *Neuroimage* 2015; **122:** 166-176.

4. Andersson JLR, Sotiropoulos SN. An integrated approach to correction for off-resonance effects and subject movement in diffusion MR imaging. *Neuroimage* 2016; **125:** 1063-1078.

5. Nir TM, Jahanshad N, Villalon-Reina JE, Isaev D, Zavaliangos-Petropulu A, Zhan L *et al.* Fractional anisotropy derived from the diffusion tensor distribution function boosts power to detect Alzheimer's disease deficits. *Magn Reson Med* 2017; **78**(6)**:** 2322-2333.

6. Mori S, Oishi K, Jiang H, Jiang L, Li X, Akhter K *et al.* Stereotaxic white matter atlas based on diffusion tensor imaging in an ICBM template. *Neuroimage* 2008; **40**(2)**:** 570-582.

7. Smith SM, Jenkinson M, Johansen-Berg H, Rueckert D, Nichols TE, Mackay CE *et al.* Tract-based spatial statistics: voxelwise analysis of multi-subject diffusion data. *Neuroimage* 2006; **31**(4)**:** 1487-1505.

8. Daducci A, Canales-Rodriguez EJ, Zhang H, Dyrby TB, Alexander DC, Thiran JP. Accelerated Microstructure Imaging via Convex Optimization (AMICO) from diffusion MRI data. *Neuroimage* 2015; **105:** 32-44.

9. Zhang Y, Brady M, Smith S. Segmentation of brain MR images through a hidden Markov random field model and the expectation-maximization algorithm. *IEEE Trans Med Imaging* 2001; **20**(1)**:** 45-57.

10. Lloyd-Jones DM, Hong Y, Labarthe D, Mozaffarian D, Appel LJ, Van Horn L *et al.* Defining and setting national goals for cardiovascular health promotion and disease reduction: the American Heart Association's strategic Impact Goal through 2020 and beyond. *Circulation* 2010; **121**(4)**:** 586-613.

11. Hayman LL, Martyn-Nemeth P. A New Metric for Promoting Cardiovascular Health: Life's Essential 8. *J Cardiovasc Nurs* 2022.

12. Lloyd-Jones DM, Allen NB, Anderson CAM, Black T, Brewer LC, Foraker RE *et al.* Life's Essential 8: Updating and Enhancing the American Heart Association's Construct of Cardiovascular Health: A Presidential Advisory From the American Heart Association. *Circulation* 2022; **146**(5)**:** e18-e43.

13. Bradbury KE, Young HJ, Guo W, Key TJ. Dietary assessment in UK Biobank: an evaluation of the performance of the touchscreen dietary questionnaire. *J Nutr Sci* 2018; **7:** e6.

14. Discacciati A, Bellavia A, Lee JJ, Mazumdar M, Valeri L. Med4way: a Stata command to investigate mediating and interactive mechanisms using the four-way effect decomposition. *Int J Epidemiol* 2018.

15. Pence BD. Growth Differentiation Factor-15 in Immunity and Aging. *Front Aging* 2022; **3:** 837575.

16. Patel AR, Frikke-Schmidt H, Bezy O, Sabatini PV, Rittig N, Jessen N *et al.* LPS induces rapid increase in GDF15 levels in mice, rats, and humans but is not required for anorexia in mice. *Am J Physiol Gastrointest Liver Physiol* 2022; **322**(2)**:** G247-G255.

17. Parchwani D, Dholariya S, Katoch C, Singh R. Growth differentiation factor 15 as an emerging novel biomarker in SARS-CoV-2 infection. *World J Methodol* 2022; **12**(5)**:** 438-447.

18. Myojin Y, Hikita H, Tahata Y, Doi A, Kato S, Sasaki Y *et al.* Serum growth differentiation factor 15 predicts hepatocellular carcinoma occurrence after hepatitis C virus elimination. *Aliment Pharmacol Ther* 2022; **55**(4)**:** 422-433.

19. de Morais Batista F, Puga MAM, da Silva PV, Oliveira R, Dos Santos PCP, da Silva BO *et al.* Serum biomarkers associated with SARS-CoV-2 severity. *Sci Rep* 2022; **12**(1)**:** 15999.

20. Ahmed DS, Isnard S, Berini C, Lin J, Routy JP, Royston L. Coping With Stress: The Mitokine GDF-15 as a Biomarker of COVID-19 Severity. *Front Immunol* 2022; **13:** 820350.

21. Agarwal N, Ramirez Bustamante CE, Wu H, Armamento-Villareal R, Lake JE, Balasubramanyam A *et al.* Heightened levels of plasma growth differentiation factor 15 in men living with HIV. *Physiol Rep* 2022; **10**(9)**:** e15293.

22. Stemmler A, Symmank J, Steinmetz J, von Brandenstein K, Hennig CL, Jacobs C. GDF15 Supports the Inflammatory Response of PdL Fibroblasts Stimulated by P. gingivalis LPS and Concurrent Compression. *Int J Mol Sci* 2021; **22**(24).

23. Muller-Heck RM, Bosken B, Michiels I, Dudda M, Jager M, Flohe SB. Major Surgical Trauma Impairs the Function of Natural Killer Cells but Does Not Affect Monocyte Cytokine Synthesis. *Life (Basel)* 2021; **12**(1).

24. Li H, Tang D, Chen J, Hu Y, Cai X, Zhang P. The Clinical Value of GDF15 and Its Prospective Mechanism in Sepsis. *Front Immunol* 2021; **12:** 710977.

25. Notz Q, Schmalzing M, Wedekink F, Schlesinger T, Gernert M, Herrmann J *et al.* Pro- and Anti-Inflammatory Responses in Severe COVID-19-Induced Acute Respiratory Distress Syndrome-An Observational Pilot Study. *Front Immunol* 2020; **11:** 581338.

26. Myhre PL, Prebensen C, Strand H, Roysland R, Jonassen CM, Rangberg A *et al.* Growth Differentiation Factor 15 Provides Prognostic Information Superior to Established Cardiovascular and Inflammatory Biomarkers in Unselected Patients Hospitalized With COVID-19. *Circulation* 2020; **142**(22)**:** 2128-2137.

27. Cao R, Zhang S, Zhang J, Miao D, Zhou H, Chen Y. Association between serum HE4 and poor periodontal health in adult women. *Clin Oral Investig* 2023.

28. Giudici KV, de Souto Barreto P, Guyonnet S, Morley JE, Nguyen AD, Aggarwal G *et al.* TNFR-1 and GDF-15 Are Associated With Plasma Neurofilament Light Chain and Progranulin Among Community-Dwelling Older Adults: A Secondary Analysis of the MAPT Study. *J Gerontol A Biol Sci Med Sci* 2023; **78**(4)**:** 569-578.

29. Casanova R, Anderson AM, Barnard RT, Justice JN, Kucharska-Newton A, Windham BG *et al.* Is an MRI-derived anatomical measure of dementia risk also a measure of brain aging? *Geroscience* 2023; **45**(1)**:** 439-450.

30. He L, de Souto Barreto P, Sanchez Sanchez JL, Rolland Y, Guyonnet S, Parini A *et al.* Prospective Associations of Plasma Growth Differentiation Factor 15 With Physical Performance and Cognitive Functions in Older Adults. *J Gerontol A Biol Sci Med Sci* 2022; **77**(12)**:** 2420-2428.

31. Wu PF, Zhang XH, Zhou P, Yin R, Zhou XT, Zhang W. Growth Differentiation Factor 15 Is Associated With Alzheimer's Disease Risk. *Front Genet* 2021; **12:** 700371.

32. Jiang WW, Zhang ZZ, He PP, Jiang LP, Chen JZ, Zhang XT *et al.* Emerging roles of growth differentiation factor-15 in brain disorders (Review). *Exp Ther Med* 2021; **22**(5)**:** 1270.

33. McGrath ER, Himali JJ, Levy D, Conner SC, DeCarli C, Pase MP *et al.* Growth Differentiation Factor 15 and NT-proBNP as Blood-Based Markers of Vascular Brain Injury and Dementia. *J Am Heart Assoc* 2020; **9**(19)**:** e014659.

34. Ebihara T, Matsubara T, Togami Y, Matsumoto H, Tachino J, Matsuura H *et al.* Combination of WFDC2, CHI3L1, and KRT19 in Plasma Defines a Clinically Useful Molecular Phenotype Associated with Prognosis in Critically Ill COVID-19 Patients. *J Clin Immunol* 2023; **43**(2)**:** 286-298.

35. Ferreira JP, Ouwerkerk W, Santema BT, van Veldhuisen DJ, Lang CC, Ng LL *et al.* Differences in biomarkers and molecular pathways according to age for patients with HFrEF. *Cardiovasc Res* 2021; **117**(10)**:** 2228-2236.

36. Li L, Yao Y, Liang J, Zhan X, Wang F, Yue C *et al.* Serum human epididymis protein 4 concentrations are associated with severity of patients with pulmonary tuberculosis. *Clin Chim Acta* 2020; **502:** 255-260.

37. Hofman VJ, Moreilhon C, Brest PD, Lassalle S, Le Brigand K, Sicard D *et al.* Gene expression profiling in human gastric mucosa infected with Helicobacter pylori. *Mod Pathol* 2007; **20**(9)**:** 974-989.

38. Bai F, Li T, Li B, Li X, Zhu L. Serum Human Epididymis Protein 4 Level is Associated with Cognitive Function in Patients with Diabetes Mellitus. *Diabetes Metab Syndr Obes* 2020; **13:** 3919-3924.

39. Lindbohm JV, Mars N, Walker KA, Singh-Manoux A, Livingston G, Brunner EJ *et al.* Plasma proteins, cognitive decline, and 20-year risk of dementia in the Whitehall II and Atherosclerosis Risk in Communities studies. *Alzheimers Dement* 2022; **18**(4)**:** 612-624.
